# Prevalence and Correlates of Alcohol Dependence Disorder among TB and HIV Infected Patients in Zambia

**DOI:** 10.1371/journal.pone.0074406

**Published:** 2013-09-17

**Authors:** Rebecca O’Connell, Nathaniel Chishinga, Eugene Kinyanda, Vikram Patel, Helen Ayles, Helen A. Weiss, Soraya Seedat

**Affiliations:** 1 Clinical Research Department, London School of Hygiene & Tropical Medicine, London, United Kingdom; 2 Zambian Aids Related Tuberculosis Project (ZAMBART), School of Medicine, Ridgeway Campus, Lusaka, Zambia; 3 Department of Nutrition and Public Health Interventions Research, London School of Hygiene & Tropical Medicine, London, United Kingdom; 4 Medical Research Council/Uganda Virus Research Institute, Unit on AIDS, Entebbe, Uganda; 5 MRC Tropical Epidemiology Group, Department of Infectious Disease Epidemiology, London School of Hygiene & Tropical Medicine, London, United Kingdom; 6 South African Research Chairs Program & MRC Stress and Anxiety Disorders Unit, Stellenbosch University, Tygerberg, Western Cape, South Africa.; 7 Department of Psychiatry, Stellenbosch University, Tygerberg, Western Cape, South Africa; Fundacion Huesped, Argentina

## Abstract

**Objectives:**

To determine the prevalence and correlates of alcohol dependence disorders in persons receiving treatment for HIV and Tuberculosis (TB) at 16 Primary Health Care centres (PHC) across Zambia.

**Methods:**

649 adult patients receiving treatment for HIV and/or TB at PHCs in Zambia (363 males, 286 females) were recruited between 1st December 2009 and 31st January 2010. Data on socio-demographic variables, clinical disease features (TB and HIV), and psychopathological status were collected. The Mini International Neuropsychiatric Interview (MINI) was used to diagnose alcohol dependence disorder. Correlates of alcohol dependence were analyzed for men only, due to low prevalence in women. Univariable and multivariable logistic regression models were used to estimate odds ratios (OR) and 95% confidence intervals (CI), using general estimating equations to allow for within-PHC clustering.

**Results:**

The prevalence of alcohol dependence was 27.2% (95%CI: 17.7-39.5%) for men and 3.9% (95%CI: 1.4-0.1%) for women. Factors associated with alcohol dependence disorder in men included being single, divorced or widowed compared with married (adjusted OR = 1.47, 95%CI: 1.00-2.14) and being unemployed (adjusted OR=1.30, 95%CI: 1.01-1.67). The highest prevalence of alcohol dependence was among HIV-test unknown TB patients (34.7%), and lowest was among HIV positive patients on treatment but without TB (14.1%), although the difference was not statistically significant (p=0.38).

**Conclusions:**

Male TB/HIV patients in this population have high prevalence of alcohol dependence disorder, and prevalence differs by HIV/TB status. Further work is needed to explore interventions to reduce harmful drinking in this population.

## Introduction

Alcohol, HIV and TB are each major contributors to global disease burden and are closely inter-related [1]. Effective intervention strategies are needed within HIV and TB treatment centres that incorporate these inter-relationships at the individual, programmatic, and structural levels. In sub-Saharan Africa, evidence suggests that alcohol impacts on HIV risk with regards to number of partners, transactional sex, violence in relation to sex, condom use, intimate partner violence and drinking environment [2] [3] [4] [5] [6] [7]. Some studies suggest a crude dose response relationship with alcohol consumption and HIV transmission [8].

Cross-sectional and qualitative studies suggest that alcohol relates to increased sexual risk taking behaviors [5,9]. It may be that risk-taking behaviors that influence alcohol use also influence other sexual risk taking, or conversely, those with HIV infection turn to alcohol [5]. However, studies do support a direct relationship between alcohol and HIV incident risk [3,10,11]. This includes a risk not just to the individual using alcohol, but also to a female sexual partner [10]. In some settings, counseling interventions may be effective in reducing this risk [12].

Alcohol use may also affect HIV progression by reducing adherence to anti-retroviral therapy (ART) [13,14]. The direct effect on disease progression on the individual level is more difficult to quantify [15]. Heavy alcohol consumption is related to faster CD4 decline in those not on ART in a US population [16] and those on ART who drink are more likely to have a detectable viral load [17]. In addition, alcohol use may affect the progression of HIV-related illness such as neurocognitive impairment [18].

HIV has exacerbated the TB epidemic, with high co-infection co-morbidity [19,20]. Despite current intervention strategies, TB continues to be a significant contributor to global health burden, prompting calls for research into social determinants and programs that address social factors in TB [21,22]. Alcohol use increases risk for TB, through overlapping risks of social and physical vulnerability, and the ability to adhere to medications [23]. Like HIV, the risk of TB and alcohol is also reflected in gender differences. For men, moderate use of alcohol is not associated with increased risk of TB, but the risk is suggested to increase with heavy drinking [24].

Measures of alcohol use are diverse, which is reflected in the heterogeneity of measurement tools across studies [8,24-27]. Drinking behaviours can be contextually and culturally specific [28]. However, it is useful to differentiate drinking patterns and disorders, as each may be linked to differing outcomes, and understanding of local behaviours will inform the potential effectiveness of interventions.

Alcohol measures aim to differentiate and describe alcohol use to identify unhealthy drinking. Criteria include the quantity of alcohol, the pattern of drinking, and behavioural and physiological consequences. The Diagnostic and Statistical Manual of Mental Disorders IV (DSM-IV) differentiates alcohol abuse from dependence, and the International Classification of Diseases-10 (ICD-10) differentiates harmful drinking from alcohol dependence [29,30]. Although the DSM-IV criteria for alcohol abuse and ICD-10 criteria for harmful drinking differ, both reflect recurrent negative consequences of alcohol use over the previous year. Alcohol dependence is defined by a constellation of symptoms and signs including cognitive and physiological, related to a strong desire for a drug [29,31]. The diagnosis for alcohol dependence shows more reliability than harmful drinking or alcohol abuse [31,32]. However, there has been recognition of overlap of diagnostic categories [32]. This is reflected in the DSM-5 which recommends ‘alcohol use disorder ‘ that encompasses abuse and dependence [33].

The Mini International Neuropsychiatric Interview (MINI) was developed to distinguish alcohol use from abuse and dependence by questionnaire, and to be compatible for diagnostic criteria of the ICD-10 [30] and the DSM IV [29]. The MINI has undergone cross-cultural validation [34], and has been used in South Africa for diagnosis and as a validation tool [35,36]. Adaptive processes for local contexts include translation and back translation together with investigation of the aptness of the test for the community [37]. However the MINI has not been validated in the Southern African context to the authors’ knowledge, although the DSM-IV and ICD-10 criteria, from which the MINI is derived, are used in routine clinical practice in Southern Africa for the diagnosis of mental health disorders.

The aims of this study are to describe and investigate prevalence of alcohol use disorder in men with HIV and/or TB diagnoses in Zambia. The inter-relationships of TB, HIV, alcohol use and gender are clear in Zambia, where the estimated population HIV prevalence is approximately 14.3% and TB prevalence is 421 per 100,000 [38] [39]. HIV prevalence is higher in urban (23.1%) than rural (10%) areas, and is higher in women than men, especially among young people. Among 15-24 year olds, the estimated prevalence is 11.3% for women, and 3.6% for men; this relationship is reversed at older ages (12.1% vs 18.6%) [38].

Alcohol use is recognized as a factor in HIV transmission in the Zambian Government’s 2009 strategy for the prevention of HIV and AIDS [40]. The Demographic and Health Survey of Zambia acknowledges the link between alcohol use and sexual and physical violence towards women: women are more likely to suffer sexual/physical violence if their partner drinks alcohol [41]. Identifying alcohol use patterns and factors associated with harmful drinking in Zambia may facilitate strategies for complementary action in HIV and TB programs.

## Methods

### Ethics Statement

Ethics approval was granted by the University of Zambia Biomedical Research Ethics Committee and the study was endorsed by the Ministry of Health in Zambia [42]. Written informed consent was obtained from eligible patients prior to participation. At the time of the study, the Zambian ethics committee were informed that the participants for inclusion in this study were 16 years and above and this was approved. A recent Bill enacted by the Zambian Parliament in 2013 has put the age of consent for medical research at 18 years and above [43].

We conducted a cross-sectional survey in 16 primary health care centres (PHCs) [42]. The TB and HIV activities in the PHCs, located in seven districts, were coordinated by the Zambia AIDS Related TB (ZAMBART) Project - a research collaboration between the University of Zambia School of Medicine and the London School of Hygiene and Tropical Medicine (LSHTM). Each PHC is staffed by primary health care physicians and includes an HIV clinic and a TB clinic and acts as the first point of entry into the referral process for the majority of TB and HIV patients.

For this study, inclusion criteria determined that participants were at least 16 years of age, and attended the TB or ART clinics of one of the PHC’s, and had started TB treatment or ART. Due care was taken to avoid recruiting the same participant at the HIV and TB Clinic. Exclusion criteria were medical or psychiatric illness that precluded capacity for consent. Eligible patients were recruited consecutively from the PHC centres from 1^st^ December 2009 to 31^st^ January 2010.

Clinic records confirming HIV and/or TB disease status were required. TB disease was defined to include pulmonary smear positive, pulmonary smear negative or extra-pulmonary TB. All patients suspected of TB were investigated by examination of sputum by microscopy and/or culture. The diagnosis of smear negative or extra-pulmonary TB without laboratory confirmation was made by a health care provider after considering indications as recommended by the World Health Organization [44].

Routine HIV testing is offered to all attendees at the PHCs. Therefore, HIV testing was routinely offered to all newly diagnosed TB patients, and all those presenting to HIV clinics prior to starting ART. Medical records were examined for evidence of the HIV test and result.

Assessment of alcohol dependence and abuse was undertaken as part of a wider study looking at screening tools for mood and mental health disorders in this population [42]. An interviewer administered MINI questionnaire was used to diagnose alcohol dependence and alcohol abuse disorder. This involved a screening question in which the participant recalled alcohol use in the past year, followed by a two-part questionnaire to define alcohol dependence or abuse, respectively. For the purposes of this analysis, the MINI was also used to define current depression. This uses a screening tool based on symptoms of the previous two weeks. If the screen is positive, the patient continues a further questionnaire.

For this study, the MINI was translated into four local languages. Community representatives then back translated, and discrepancies were resolved through an informal committee consensus. Next, translations were piloted and any disagreement was discussed until a final version was agreed upon. Sixteen lay research assistants and 10 mental health care workers were recruited from the study communities and trained on how to obtain informed consent from the participants and on the data collection process.

After informed consent, recruitment and interview took place on the same day. Sociodemographic variables were collected through interviewer-administered questionnaires. Data were double-entered to an Access database.

### Statistical methods

**Figure 1:**
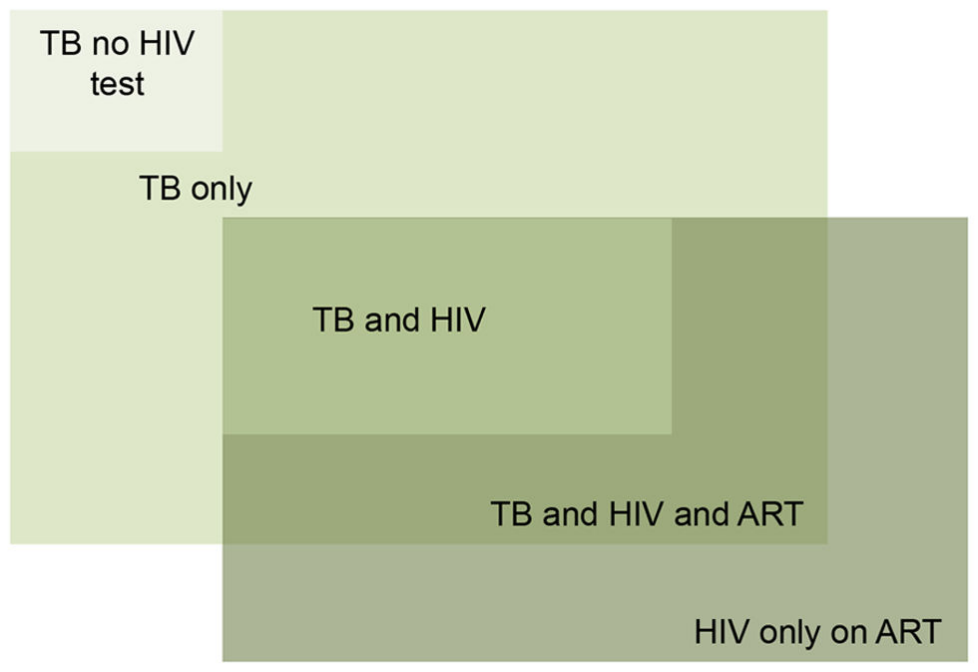
Schematic of clinical categories of participants.

All analysis was performed using STATA 11. The main outcome was alcohol dependence. The three main clinical diagnostic groups included TB disease, TB disease with HIV (TB/HIV positive co-infected), and HIV infection only (no TB disease). Further categorization included a group of participants with TB who had not had an HIV test. Also, TB/HIV positive co-infected patients were considered as to whether they were taking antiretroviral therapy (Figure 1).

Due to potential within-PHC clustering, STATA survey commands and general estimating equations (GEE) logistic regression were used to estimate odds ratios (OR) and 95% confidence intervals (CI). Participants were not recruited considering probability of selection; therefore post study weighting was performed. We used a pragmatic approach: weighting was designed to take into account the combined size of the TB and HIV clinic in each PHC. Weights created were proportional to the inverse probability of being selected.

For multivariable analysis, a hierarchical approach was used to investigate sociodemographic and clinical factors [45]. Variables considered to have a strong *a priori* hypothesis for confounding included age, and this was included in all models, together with those with p-value < 0.2 on univariable analysis using the Wald test. Sociodemographic variables were added in order of considered hierarchical importance in proximate and ‘upstream’ influence on the outcome alcohol dependence. After the addition of clinical categories as the first variable in the model as the most proximate factor, intermediate factors were incorporated, including social variables such as marital status and religion. Finally, more distal factors including those reflecting socioeconomic status such as education, employment and rural or urban environment were included. The final model incorporated those variables with evidence for an independent association with alcohol dependence (Wald p value < 0.1), and those with *a priori* hypothesis and confounders of alcohol dependence and the clinical variables.

## Results

**Figure 2:**
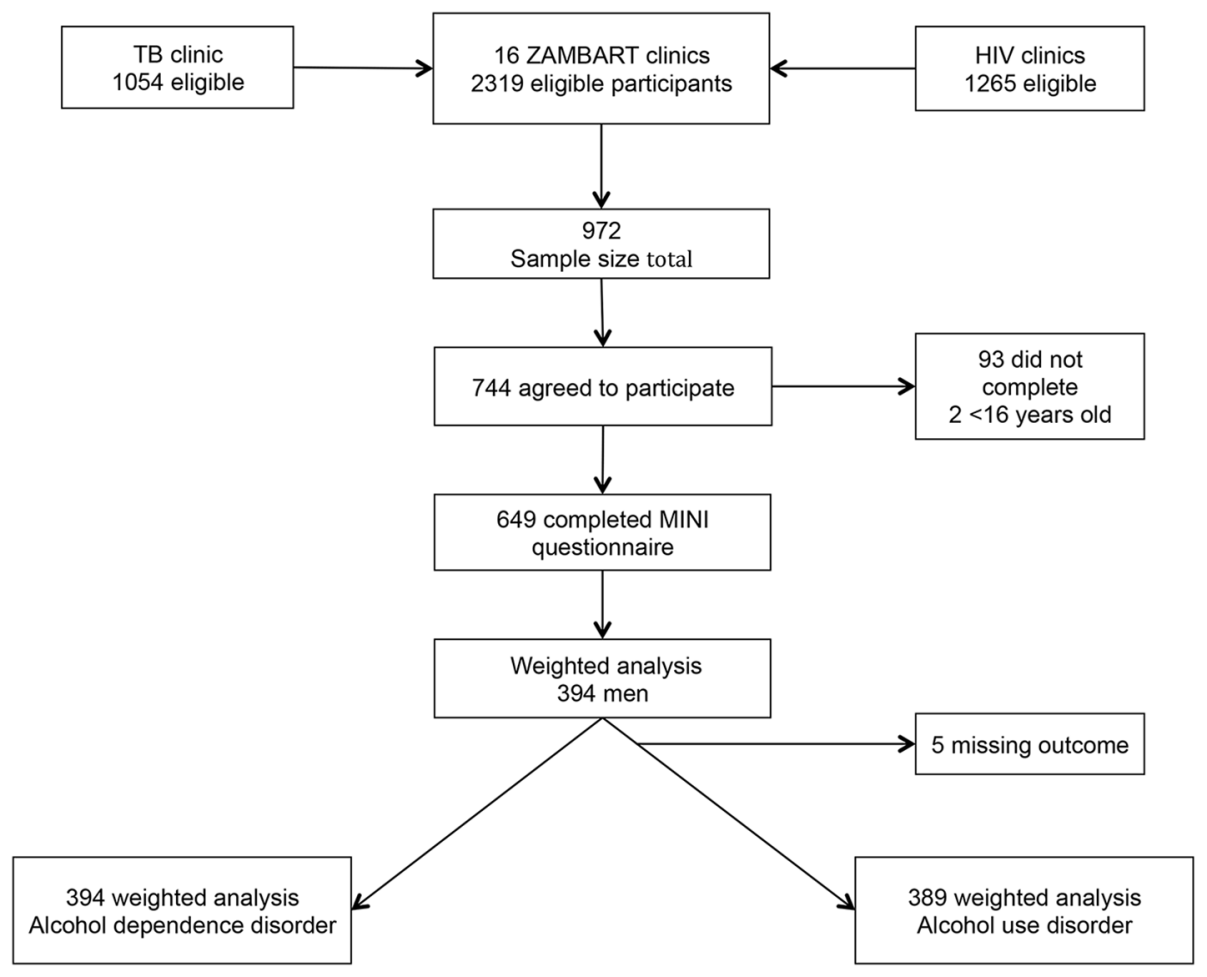
Eligibility and participant inclusion.

During the study period, 2319 patients attended the TB and/or HIV clinics. Of these, 744 (32.1%) agreed to participate at the screening stage, and 649 (28%) completed the MINI questionnaire (Figure 2). Reasons for not participating were not formally recorded but included: time constraints; confidentiality concerns; and women needing to seek permission from their husbands but not returning to complete the study. There were no reported exclusions due to alcohol intoxication.

Among the 649 participants, 394 (60.7%) were men and 255 (39.3%) were women. Of the 394 men, 107 (27.2%) had a diagnosis of alcohol dependence and 20 (5%) a diagnosis of alcohol abuse. Of the 255 women, 10 (3.9%) had a diagnosis of alcohol dependence disorder and 5 (1.9%) a diagnosis of alcohol abuse. Subsequent analyses focus on the 394 males in the study population.

The socio-demographic characteristics of the 394 men are shown in Table 1. The largest PHCs were those in the capital city Lusaka accounting for 62.3% of the men in this study, only 7.9% of the men were from rural PHCs. Unemployment was reported in 175 (44.3%) of the men. Over half (55.3%) were in current marriages, with 28.7% describing themselves as single and 11.4% divorced. The majority had some formal education, with 43.2% attending grade 8 to 12, and 12.6% going to college. Most participants described themselves as members of Christian churches (90.9%), within which there was frequent self-identification with unique, single church Christian organizations.

**Table 1:** Sociodemographic characteristics of the study male population.

**Variable**	**Category**	**N**	**% (CI**)
Gender		394	60.7 (52.4,68.5)
Age	16-29	125	31.7 (28.4,35.3)
	30-34	91	23.0 (18.9,27.7)
	35-40	68	17.2 (12.5,23.2)
	>40	99	25.2 (21.6,29.2)
	Missing	11	2.8 (2.2,3.7)
Language	English	33	8.2 (3.1,20.1)
	Tonga	19	4.8 (1.3,18.8)
	Bemba	125	31.7 (13.5,57.9)
	Lozi	20	5.2 (0.8,27.6)
	Nyanja	197	50.0 (24.2,75.9)
Education	No school	21	5.5 (3.1,9.4)
	Grade 0 to 7	145	36.7 (31.4,42.4)
	Grade 8 to 12	171	43.2 (37,49.9)
	College	49	12.6 (8.9,17.6)
	Missing	8	1.9 (0.4,8.2)
Work	Work or other activity	219	55.7 (47.1,63.9)
	No work	175	44.3 (36.1,52.9)
Marital status	Married	218	55.3 (46.6,63.7)
	Single	113	28.7 (20.7,38.3)
	Divorced	45	11.4 (6.7,18.7)
	Widowed	18	4.6 (2.8,7.4)
Religion	Catholic	94	23.9 (18.5,30.4)
	Protestant	107	27.2 (22.4,32.7)
	Muslim	5	1.3 (0.5,3.2)
	Seventh Day Adventist	49	12.4 (7.3,20.1)
	Jehovah Witness	30	7.7 (4.4,13.3)
	Pentecostal/ Evangelical	73	18.6 (13.8,24.6)
	No religion	22	5.5 (2.5,11.6)
	Other	12	3.0 (1,8.9)
	Missing	2	0.5 (0.1,4.0)
Environment 1	Rural	31	7.9 (2.1,25.9)
	Urban	363	92.1 (74.1,97.9)
Environment 2	Provincial	149	37.7 (12.9,71.1)
	Capital	245	62.3 (28.9,87.1)

Clinical characteristics of the participants are shown in Table 2. Over 90% (355/394) of participants had TB and over half (205/394) were HIV positive. Of those HIV positive, 50.9% were taking ART. Further clarification of clinical diagnosis includes: 16.9% (66/394) had TB but did not have an HIV test result; 31.2% (123/394) had TB and were HIV negative; 9.8% (39/394) had HIV only and were taking ART; 25.5% (101/394) had HIV/ TB co-infection but were not taking ART; 16.6% (65/394) had HIV/TB co-infection and were taking ART.

**Table 2:** Clinical characteristics of the male participants.

**Variable**	**Category**	**Weighted male n**	**Prevalence CI %**
HIV status	Not tested	66	16.8 (14.3,19.7)
	Negative	123	31.2 (25.0,38.1)
	Positive	205	52.0 (46.6,57.4)
TB status	No TB	39	9.8 (4.1,21.9)
	TB	355	90.2 (78.1,95.9)
HIV treatment status	No	101	49.1 (39.9,58.4)
	Yes	104	50.9 (41.6,60.1)
TB retreatment	No	229	64.5 (54.1,73.7)
	Yes	120	33.8 (23.4,46.1)
	Missing	6	1.7 (0.4,6.4)
Clinical diagnosis	HIV test unknown, TB	66	16.9 (14.3,19.7)
	HIV negative, TB	123	31.2 (25.0,38.1)
	HIV/ TB co-infection no ART	101	25.5 (20.6,31.2)
	HIV/ TB co-infection on ART	65	16.6 (11.9,22.7)
	HIV positive only, no TB	39	9.8 (4.1,21.9)
CD4	<=200	44	42.5 (27.0,59.5)
(for those on ART)	>200	23	22.1 (15.2,31.1)
	Missing	37	35.0 (20.1,54.4)
Alcohol use disorder	No	262	66.4 (54.8,76.3)
	Yes	127	32.3 (22.4,44.0)
	Missing	5	1.3 (0.4,4.7)
Alcohol abuse	No	369	93.6 (89.1,96.3)
	Yes	20	5.0 (2.2,11.2)
	Missing	5	1.4 (0.4,4.7)
Alcohol dependence	No	287	72.8 (60.5,82.3)
	Yes	107	27.2 (17.7,39.5)
	Missing	0	0
Current depression	Yes	43 (11)	11.0 (8.3,14.5)

On univariable analysis, alcohol dependence was associated with younger age (p-trend=0.01; Table 3). Those who were currently single were more likely to be alcohol dependent than those who were married (OR=1.69; CI: 1.20-2.35). Employment status was associated with alcohol dependence i.e. those not in work were more likely to have alcohol dependence (OR=1.36; 95% CI: 1.11-1.68). Those who identified as Seventh Day Adventists (SDA) had a lower prevalence of alcohol dependence than Roman Catholics (OR=0.46; 95% CI: 0.32-0.66).

**Table 3:** Univariable analysis: associations of prevalence of alcohol dependence.

**Variable**	**Category**	**Alcohol Dependence n/N**		**Prevalence of Alcohol Dependence % (CI**)	**OR (CI**)	**p value**
Gender	Male		107/394	27.2 (17.7,39.5)		
Age	16-29		41/125	33.0(19.3,50.3)	1	
	30-34		26/93	29.0 (20.7,39.1)	0.83 (0.50,1.40)	
	35-40		14/68	21.1 (9.7,40)	0.62 (0.26,1.48)	
	>40		23/97	22.9 (12.1,39.2)	0.60 (0.38,0.95)	<0.01
	Missing		3/11	24.4 (17.7,39.5)	trend	<0.01
Education	No school		7/21	33.3 (14.5,59.5)	1	
	Grade 0 to 7		43/145	29.8 (15.7,49.2)	0.92 (0.33,2.60)	
	Grade 8 to 12		44/171	25.7 (17.6,35.9)	0.77 (0.19,3.09)	
	College		13/50	25.6 (11.9,46.7)	0.70 (0.40,1.21)	0.23
	Missing		1/7	6.5 (0.7,40.6)		
Language	English		12/33	35.7 (11.0,71.3)	1	
	Tonga		4/19	20.2 (11.4,33.4)	0.67 (0.15,3.02)	
	Bemba		29/125	23.5 (14.8,35.2)	0.84 (0.13,5.45)	
	Lozi		4/20	18.3 (12.7,25.7)	0.89 (0.17,4.80)	
	Nyanja		59/197	29.8 (14.5,51.7)	1.50 (0.37,6.41)	0.36
Employment	Employed or other occupation		5/6219	25.5 (17,3 6.4)	1	
	Unemployed		51/175	29.4 (17.7,44.7)	1.36 (1.11,1.68)	0.004
Marital status	Currently married		48/218	22.0 (14.2,32.6)	1	
	Single/ divorced/ widowed		59/176	33.7 (21.9,47.9)	1.69 (1.21,2.35)	0.002
Religion	Catholic		23/94	24.7 (12.3,43.4)	1	
	Protestant		28/107	26.3 (18.2,36.2)	1.13 (0.56,2.27)	
	SDA		7/49	13.7 (5.1,32.0)	0.46 (0.32,0.66)	
	JW		12/30	39.4 (18.6,64.8)	1.75 (1.22,2.51)	
	Pentecostal/ Evangelical		22/73	30.1 (18.4,45.2)	1.41 (0.89,2.24)	
	No religion		10/22	46.3 (12.6,83.8)	2.57 (0.88,7.52)	
	Other		5/17	31.2 (7.4,72.0)	1.56 (0.18,13.8)	<0.001
	Missing		0/2	0		
Environment 1	Rural		8/31	26.8 (19.2,36.1)	1	
	Urban		99/363	27.3 (17.1,40.6)	1 (0.49,2.02)	1
Environment 2	Provincial		33/149	21.9 (14.7,30.5)	1	
	Capital		75/245	30.5 (16.7,48.9)	1.80 (0.73,4.44)	0.2
HIV status	Negative		41/123	33.4 (22.7,46.2)	1	
	Positive		43/205	21.1 (12.1,34.3)	0.61 (0.34,1.11)	
	Not tested		23/66	34.7 (17.5,57.2)	1.20 (0.70,2.08)	0.15
TB status	No treatment		6/39	14.1 (6.0,29.6)	1	
	Treatment		102/355	28.7 (18.1,42.2)	2.31 (0.67,7.97)	0.18
HIV treatment	No		25/101	25.2 (13.8,41.4)	1	
	Yes		18/104	17.2 (9.0,30.5)	0.62 (0.33,1.18)	0.15
TB retreatment	No		63/229	27.3 (16.2,42.2)	1	
	Yes		36/120	29.8 (15.5,49.5)	1.28 (0.60,2.75)	0.73
	Missing		4/6	59.7 (13.7,93.3)		
Clinical diagnosis	HIV negative, TB		41/123	33.4 (22.7,46.2)	1	
	HIV test unknown, TB		23/66	34.7 (17.5,57.2)	1.19 (0.69,2.06)	
	HIV/ TB co-infection no HIV treatment		25/101	25.2 (13.8,41.4)	0.76 (0.42,1.35)	
	HIV/ TB co-infection on HIV treatment		12/65	19.1 (8.4,37.5)	0.53 (0.26,1.10)	
	HIV positive only		5/39	14.1 (6.0,29.6)	0.39 (0.11, 1.26)	0.33
Depression	No		97/351	27.7 (18.0,40.1)	1	
	Yes		10/43	23.3 (11.1,42.7)	0.73 (0.38,1.38)	0.33

There was a suggestion of difference in alcohol prevalence according to clinical diagnostic category, but univariable analysis did not support statistical differences between patient populations. Alcohol dependence was more prevalent among those with TB compared to those with HIV only (OR=2.31; CI = 0.67–7.97). Among HIV positive patients, alcohol dependence was less common among those on ART than those not on ART (OR=0.62; 95% CI: 0.33-1.18). There was no association between alcohol dependence and being on TB retreatment (OR=1.28; 95% CI: 0.60-2.75).

Multivariable analyses (Table 4) showed that, with TB infection only as reference, those with HIV only on ART were least likely to have alcohol dependence (OR=0.43; 95% CI: 0.15-1.29) followed by those with TB and HIV on ART (OR=0.63; 95% CI: 0.23-.72). However, there was not strong evidence to support a true difference between clinical groups. Religion remained independently associated with alcohol dependence. Other proximate risk factors somewhat associated with alcohol dependence were being single compared with currently married (OR=1.47; 95% CI: 1.00-2.14), and being out of work compared with being employed (OR=1.30; 95% CI: 1.01-1.67).

**Table 4:** Multivariable analysis: associations of prevalence of alcohol dependence.

**Variable**	**Category**	**OR**	**CI**	**p value**
Clinical categories	TB only	1		0.38
	TB HIV unknown	1.17	0.62,2.22	
	TB and HIV no ART	0.95	0.38,2.41	
	TB/ HIV and ART	0.63	0.23,1.72	
	HIV only and ART	0.43	0.15,1.29	
Age	16-29	1		0.78
	30-34	0.97	0.45,2.06	
	35-40	0.75	0.20,2.78	
	>40	0.91	0.61,1.34	
	Missing	0.61	0.16,2.34	
Marital status	Married	1		0.05
	Single, divorced, widowed	1.47	1.00,2.14	
Employment	Employed or other	1		0.04
	Unemployed or other	1.30	1.01,1.67	
Religion	Catholic	1		<0.01
	Protestant	1.32	0.68,2.57	
	SDA	0.58	0.37,0.89	
	JW	1.79	0.93,3.45	
	Pentecostal/ Evangelical	1.73	1.00,2.99	
	No religion	2.51	1.00,6.27	
	Other	1.79	0.14,23.3	

## Discussion

The prevalence of alcohol dependence disorder was high among men with TB disease and/or HIV (27.2%). Women with TB disease and/or HIV had a low prevalence of alcohol dependence disorder (3.9%). This striking gender difference is in keeping with other study findings of alcohol use in Zambia and other sub Saharan African countries [27,28,46-48]. For example, in a South African study among HIV patients, male alcohol dependence was 22.7%, while female alcohol dependence was 4.7% [49]. In a primary health care setting (non-HIV/TB) in Uganda, the prevalence of alcohol dependence using the DSM diagnostic criteria, was 14.9% for males and 4.9% for females [50]. Although men tend to have a higher prevalence of alcohol use disorders than women, the consequences are also felt by women who are at increased risk of HIV transmission and partner violence [5,51].

There are no general population studies which have used the MINI in Zambia, but in a population survey conducted in 2004 in which alcohol use was self-reported, 9.5% of the men reported as infrequent heavy drinkers, compared with 1.2% of women; and 2.6% of men as frequent heavy drinkers versus 0.5% of women [46]. Although not directly comparable due to different definitions of alcohol use, the prevalence of problem drinking in the general population seems lower than our study population. A study looking at alcohol use in Zambian HIV serodiscordant couples found similar gender disparity [52].

While the prevalence of alcohol dependence is clearly higher in women than men in our study, gender impacted participation in the study and social desirability may impact on willingness to disclose to health care professionals. Some women wished to seek clarification from a partner regarding participation, and then did not return. Also, women may engage in alcohol drinking patterns associated with increased risk behaviour, but not meeting diagnostic criteria for abuse or dependence [53].

In assessing evidence for the relationship between alcohol, HIV and TB, comparison between studies is complicated by heterogeneity of measures of alcohol use [6,9,47]. Further, alcohol use can be culturally and context specific, relating to local and particular consumption patterns, complicating further comparative analysis [54] [55]. The advantage of using DSM-IV criteria for alcohol dependence in our study, as measured by the MINI, is that it is considered to be more robust and reproducible across cultural contexts than other methods [31].

We also found differing prevalence of alcohol dependence in each clinical HIV/TB category, although this is not statistically significant. The prevalence is lowest in those with HIV only, and highest in those with TB only. As with all cross-sectional studies, limitations prohibit defining causal associations. In other studies investigating TB, the association with chronic heavy alcohol use is clear, and this may be the case in this study. Alternatively, it may be that those who have alcohol use disorder are not offered ART, and/or those starting ART are advised not to drink. In addition, those on ART may change drinking patterns due to the desire to become well with ART.

Alcohol dependence was more prevalent than abuse in this study, as measured by the MINI. Drawing a distinction between the excess chronic harmful alcohol use of alcohol dependence and ‘acute’ harmful alcohol use may be necessary in aiding design of programmes to address alcohol reduction strategies in differing clinical contexts. Different drinking disorders, patterns and contexts may have differing consequences [5,28]. In diagnosing alcohol use disorder using the MINI questionnaire, dependence precludes a diagnosis of abuse. However, studies from the USA suggest the two diagnoses may co-occur: abuse patterns may occur in those with dependence; those with abuse may develop dependence [56,57]. ‘Acute alcohol’ intake may be associated with HIV risk, whereas dependence and long-term use may be associated with deleterious effects on health, increasing susceptibility to TB, and both patterns could impact on adherence.

Social factors associated with alcohol dependence in this study included marital status and occupation status. Those who were not married were more likely to be diagnosed with alcohol dependence, as were those unemployed. In this study, the group with active occupations is heterogeneous, including students and the retired and further differentiation is necessary to clarify associations. Direction of association in both marital status and employment cases is difficult to determine. Comparison with other studies is difficult, as dependence may have different sociodemographic associations than other measures of alcohol use, or alcohol abuse. Suliman et al found such differing social factors associated with differing stages of alcohol intake and alcohol use disorders in looking at individuals’ transitions from any alcohol use to alcohol abuse and dependence [48]. For example, this may in part explain why Kullgren et al found no association with occupation for alcohol dependence using the CAGE and DSM IV criteria [50].

Religion of choice, or not having a religion, appears to be associated with alcohol use disorder. Seventh Day Adventists were least likely to have diagnosis of alcohol use disorder and those with no stated religion had the highest prevalence. Other studies have found adherence to religion to impact on alcohol use, and religious beliefs may influence complete abstention [58]. However, this may also represent lack of willing to disclose alcohol use and under-reporting may occur [59]. The diverse responses and self-identification with independent churches further complicates analysis. Due to these constraints, and small numbers of participants in each religious category, it is difficult to draw further conclusions as to how alcohol use disorder determined in our study could relate to TB and HIV outcomes according to religion in this population.

Despite known associations of alcohol as a risk for TB and a risk to adherence to TB medications, alcohol dependence was not associated with TB retreatment in this analysis. It may be that survival in that group is poor, and they are therefore not represented. In addition, those with alcohol use disorders may not attend health services, and therefore are not included. As this applies to only a few participants, further exploration of any association or this group is beyond the scope of this study.

However, also excluded are those with issues of attendance to care. Retention in all stages of care is an ongoing problem for HIV health care delivery [60]. There are no data on loss to follow up in our cross-sectional survey. Alcohol dependence could be considered as a factor for both these aspects of HIV and TB health care delivery. Routine use of alcohol use measures would be useful in both groups to investigate the impact of alcohol use disorders on health centre attendance and retention in care.

Alcohol use has a clear impact on risk behaviour, on TB and HIV clinical outcomes, and on adherence to treatment. It seems plausible that alcohol impacts on attendance at health centre settings and retention in care. The diversity of alcohol measures is a barrier to interpreting the impact of alcohol on HIV and TB. Therefore, robust culturally validated yet standardized screening tools could assist in identifying harmful behaviour linked to alcohol use, and therefore where interventions could be achieved.

In HIV, there is some evidence from Sub Saharan settings to suggest counselling interventions can impact alcohol-related risk taking behaviour [61], and can encourage alcohol reduction [62]. Randomised controlled trials of interventions in the TB and HIV clinic setting would help ascertain best practice for sustainable alcohol harm reduction interventions for different harmful drinking patterns.

Our study had some methodological limitations. The survey design did not take into account the size of clinic, and therefore the probability of selection of each participant. We minimized selection bias in the analysis using post-hoc weighting. It was not possible to obtain full clinical data for participants.

As discussed, evidence from studies suggests in those with alcohol related disorders there is an association with poorer outcomes in both HIV and TB [13,17,63]. However, in this study it was not possible to comment on severity of illness and alcohol dependence, as this information was not collected. The CD4 count was only measured on those in the ART programme, and so there was insufficient CD4 data to assess whether there was more advanced HIV with alcohol dependence. The clinical type of TB, and severity of TB illness was not available.

To conclude, alcohol dependence is highly prevalent in this population, especially among males, but interventions for alcohol disorders are under-investigated in the lower income setting [25]. The TB and HIV population in our study is typical of the TB and HIV patients one would find in PHC settings in Zambia. However, our findings on alcohol dependence may not be generalisable to the Zambian population, nor to TB/HIV patients seeking treatment in other PHCs. The survey results contain a suggestion that there may be differing prevalence according to whether there is TB, HIV and/or ART and this may represent real different alcohol use patterns. If the differing prevalences are representative, this suggests incorporating alcohol use screening into HIV/TB programs may help identify those at risk and interventions could be targeted according to clinical need, and complement existing TB and HIV programs.
